# Methodological evolution of potato yield prediction: a comprehensive review

**DOI:** 10.3389/fpls.2023.1214006

**Published:** 2023-07-26

**Authors:** Yongxin Lin, Shuang Li, Shaoguang Duan, Yanran Ye, Bo Li, Guangcun Li, Dianqiu Lyv, Liping Jin, Chunsong Bian, Jiangang Liu

**Affiliations:** ^1^ State Key Laboratory of Vegetable Biobreeding, Institute of Vegetables and Flowers, Chinese Academy of Agricultural Sciences, Beijing, China; ^2^ College of Agronomy and Biotechnology, Southwest University, Chongqing, China; ^3^ Seeds Development, Syngenta Jealott’s Hill International Research Centre, Bracknell, United Kingdom

**Keywords:** yield prediction, potato, precision agriculture, remote sensing, crop growth model

## Abstract

Timely and accurate prediction of crop yield is essential for increasing crop production, estimating planting insurance, and improving trade benefits. Potato (*Solanum tuberosum* L.) is a staple food in many parts of the world and improving its yield is necessary to ensure food security and promote related industries. We conducted a comprehensive literature survey to demonstrate methodological evolution of predicting potato yield. Publications on predicting potato yield based on methods of remote sensing (RS), crop growth model (CGM), and yield limiting factor (LF) were reviewed. RS, especially satellite-based RS, is crucial in potato yield prediction and decision support over large farm areas. In contrast, CGM are often utilized to optimize management measures and address climate change. Currently, combined with the advantages of low cost and easy operation, unmanned aerial vehicle (UAV) RS combined with artificial intelligence (AI) show superior potential for predicting potato yield in precision management of large-scale farms. However, studies on potato yield prediction are still limited in the number of varieties and field sample size. In the future, it is critical to employ time-series data from multiple sources for a wider range of varieties and large field sample sizes. This study aims to provide a comprehensive review of the progress in potato yield prediction studies and to provide a theoretical reference for related research on potato.

## 1 Introduction

Global food security is encountering significant challenges from climate change and increasing resource competition (Godfray et al., 2010). Potato (*Solanum tuberosum* L.), a tuberous crop, is cultivated worldwide due to its stable and high yield, wide adaptability, and complete nutritional composition. Furthermore, it is a pivotal crop for realizing the United Nations (UN) Sustainable Development Goals (SDGs). Yield of potato and other crops is determined interactively by genotype (G), environment (E), and management practices (M) (Cooper et al., 2021). Analysis and modeling of key parameters can effectively predict crop yield, providing crucial guidance and decision support for various stakeholders, such as farmers, policy makers, and agribusinesses. In addition, these predictions substantially impact optimizing planting structure, optimizing trading policies, allocating resources efficiently, and conducting precision management.

There have been significant strides in both theoretical and practical aspects of predicting potato yield. Early on, such prediction relied on field sampling, whereby the number and weight of potatoes per unit area were measured to calculate the yield (Dyke and Avis, 1953). Other agronomic traits in subsequent studies, including petiole potassium content (Holm and Nylund, 1978), also served as useful indicators for potato yield. Nevertheless, these destructive methods require substantial labor for field sampling and do not provide complete spatial or temporal coverage.

Remote sensing (RS) has emerged as a popular tool in crop phenotyping (Araus and Cairns, 2014), growth monitoring (Liu et al., 2021), and yield prediction (Ma et al., 2021), attributed to it being non-destructive, high-throughput, and having large spatial coverage. In 1974, the Large Area Crop Inventory Experiment (LACIE) program, which showed the possibility of RS for yield prediction for the first time, was used to assess wheat acreage in the United States, Canada, and the former Soviet Union combined with Landsat (MacDonald et al., 1975). In 1977, the LACIE accurately predicted a declining trend in spring wheat production in the Soviet Union with precision of 90% leading to a positive impact on the United States economy (Hill et al., 1980). From 1980 to 1986, multiple departments involved in the LACIE program collaborated in the Agriculture and Resources Inventory Surveys Through Aerospace Remote Sensing (AgRISTARS) initiative (Doraiswamy et al., 1979). This effort aimed to predict yield for eight crops (not including potato) within the United States and other countries worldwide. Satellite-based potato yield prediction commenced later in the 1980s. Potato acreage was estimated to predict production in Canada by Landsat (Ryerson et al., 1985). In 1987, the European Union proposed the Monitoring Agricultural Resources (MARS) project, which utilized satellite and aerial imagery to continuously monitor the planting area and growth status of several staple crops including potatoes (Van der Velde and Nisini, 2019). It also provided timely prediction results of crop yields for the European Union.

Although satellites have the advantage of covering large areas of farmland, they are greatly affected by the revisit interval and low resolution. To address the growing demand for site-specific crop monitoring and yield prediction among farmers, research into proximal RS technologies such as unmanned aerial vehicle (UAV) and ground-based RS has increased rapidly. In contrast to satellites, UAVs possess tremendous potential for site-specific phenotype acquisition, yield prediction, and precision management, which is attributed to their low cost, convenience, and high spatial resolution (Yang G. et al., 2017). Additionally, ground-based methods are increasingly being utilized for more detailed phenotypic analyses in a variety of specific scenarios. In general, RS is capable of rapidly monitoring fields without damaging them. However, it employs empirical modeling methods most of which lack a robust mechanism.

Crop growth model (CGM) aim to describe the process of potato development before harvest. POTATO (Ng and Loomis, 1984) is the first CGM for potato with a complete mechanism. Between the 1990s and early 2000s, potato CGM became more comprehensive with the incorporation of additional parameters, such as water and nitrogen modules (Tang et al., 2021). During this period, various models were developed, including DSSAT-SUBSTOR (Ritchie et al., 1995) and LINTUL-NPOTATO (Van Delden et al., 2003). Although these models are mechanistic and have high precision, they require a substantial number of input parameters. Furthermore, the calibration and validation of these models rely on ground-truth data, which can be laborious to acquire.

During the 2010s, sensor technology, machine learning (ML), digital image analysis, and data mining techniques developed rapidly. Additionally, mechanisms underlying potato growth and development, yield quality formation principles, and interactions between crop-environment-management measures were better understood, encouraging improvement in theories of potato yield prediction.

Yield prediction requires multidisciplinary knowledge at the intersection of agronomy, meteorology, statistics, economics, and computer science. Several studies have reviewed the advancements made in yield prediction for diverse crops such as rice (dela Torre et al., 2021) and maize (Tandzi and Mutengwa, 2020). In contrast, yield prediction for potato differs from other major crops because its edible part is located belowground. Currently, there is no comprehensive literature survey of potato yield prediction due to complex model types and application scenarios. To tackle these issues, this paper provides an overview of the advancements in and prospects for potato yield prediction. First, we present a summary of the commonly used methodologies and compare them. Second, after thoroughly evaluating the existing methods, we envision the future development of potato yield prediction. This review comprehensively evaluates the progress made in potato yield prediction and provides the corresponding theoretical references.

## 2 Literature survey

**Figure 1:**
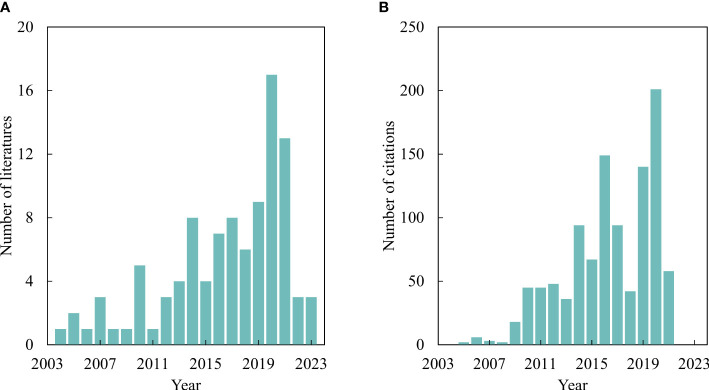
Literature counts **(A)** and citations **(B)** for potato yield production since 2003 to 2023.

A total of 276 articles including the keywords “potato”, “yield or production or output”, and “estimat* or forecast* or predict* or simulat*” were identified in the Web of Science™ database (Clarivate Analytics) through January 11, 2023. To encompass a broader scope of relevant studies, we also conducted a literature survey with the abovementioned keywords on the Scopus database and retrieved 152 publications. After eliminating duplicates and irrelevant studies, 160 publications were included in this study. As depicted in **Figure 1A**, the number of pertinent studies has progressively increased since the 2010s. Furthermore, **Figure 1B** demonstrates a growing number of annual citations for these publications, indicating increased interest in this research domain.

**Figure 2:**
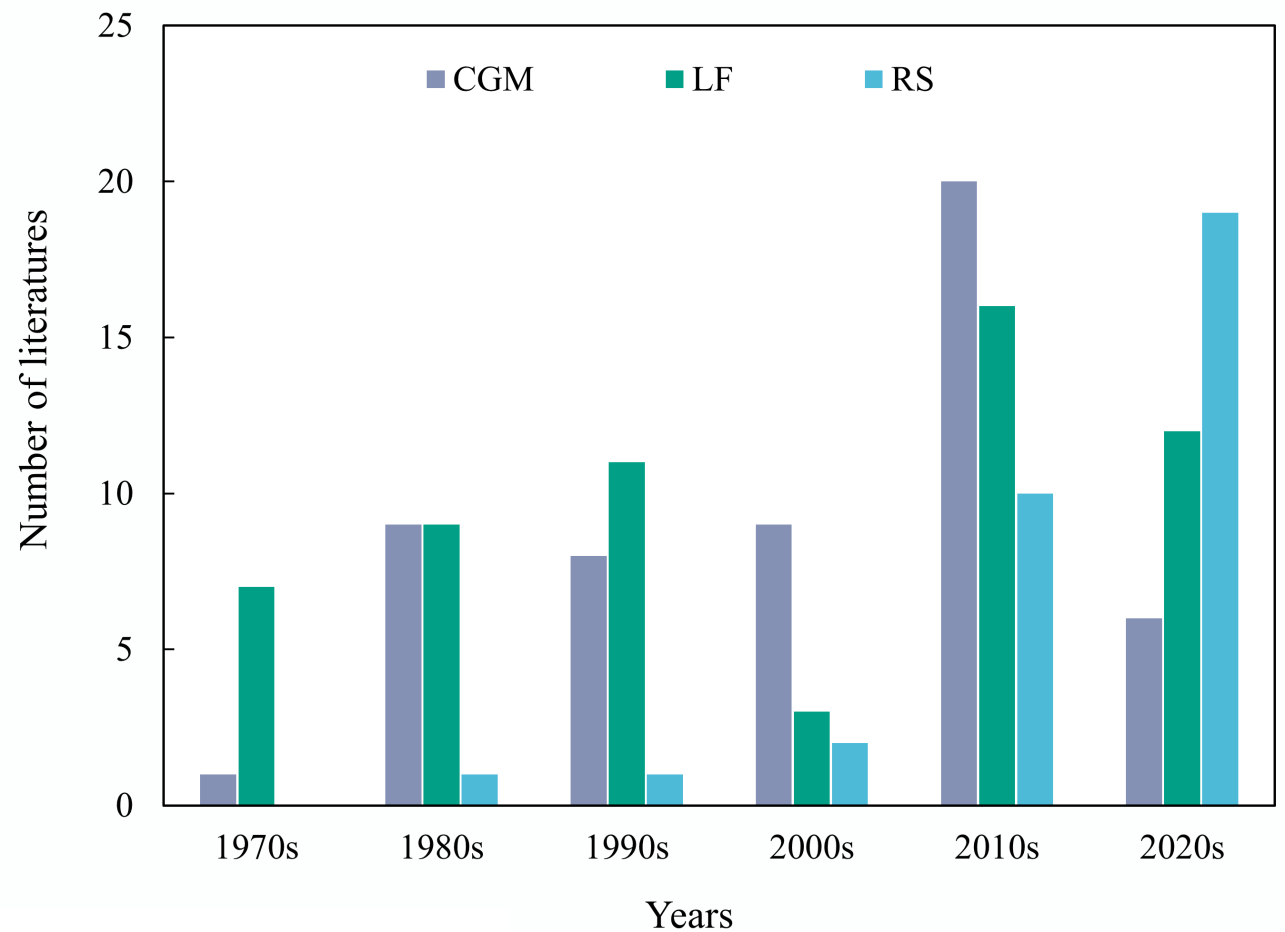
Distribution of strategies for potato yield prediction using CGM, RS, and LF-based methods since 1970s to 2020s.

**Figure 3:**
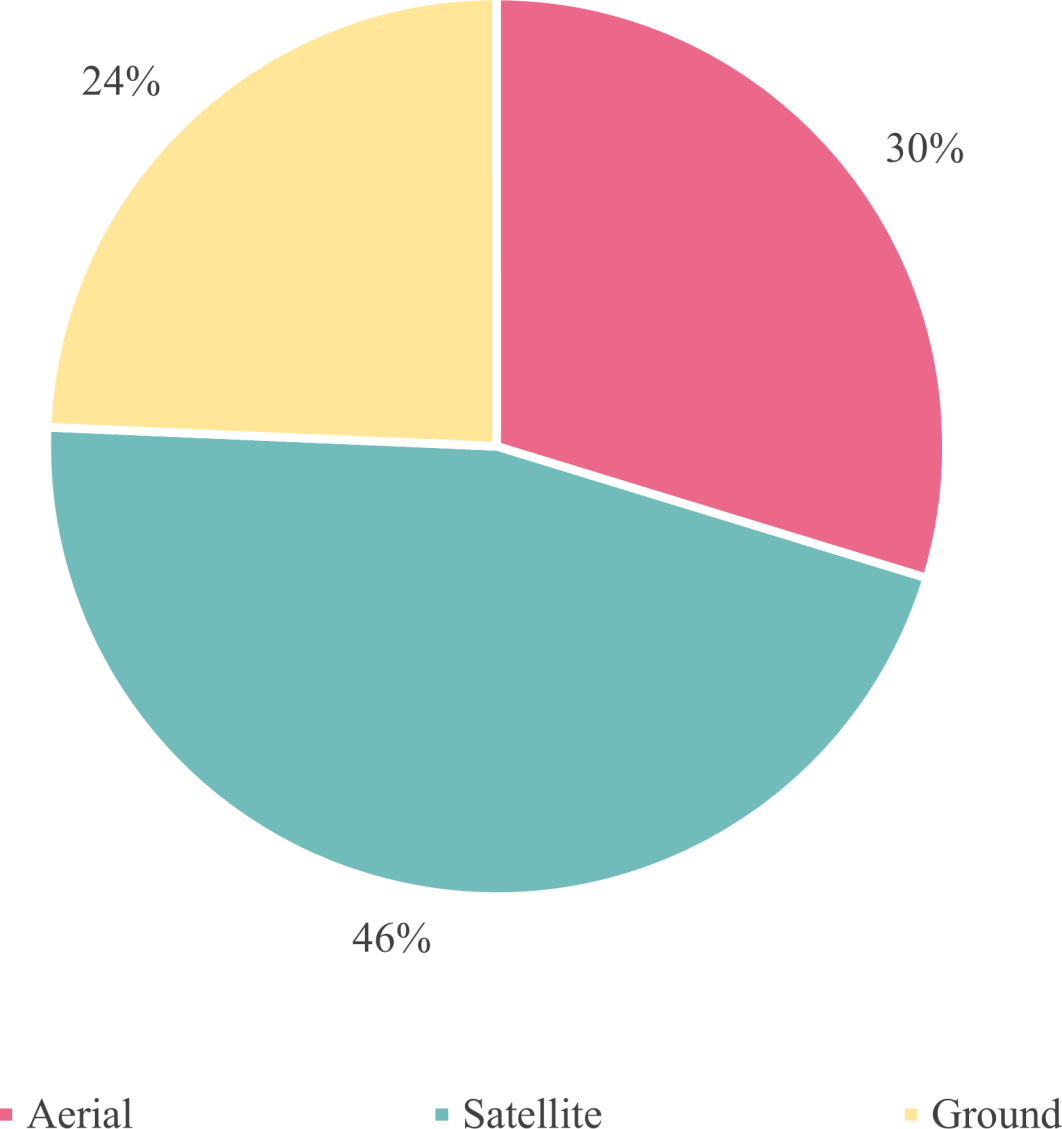
Distribution of RS Platforms adopted for potato yield prediction.

Through literature review, we classified studies on potato yield prediction into three categories including methods based on RS, CGM, and yield limiting factor (LF). Methods with LF include those based on agronomic and environmental parameters. **Figure 2** displays the numbers of the three approaches in potato yield prediction studies over the past 50 years. Initially, RS and CGM were less used for potato yield prediction. CGM-based methods have a long history with the key period of research and development occurring in the 1980s and 1990s. Since the 21st century, CGM have been widely applied, with research efforts focused on the parameterization of models under various conditions. The CO_2_ response module is integrated with climate models to assess the impact of climate change on future yields. With the development of advanced information technology, RS-based methods have emerged in the study of crop yield prediction, utilizing next-generation sensors, UAVs, and ML algorithms. For instance, in 2020, there were 17 publications relating to potato yield prediction, of which ten were dedicated to RS-based methods. These publications contain RS-based yield prediction at multiple carrying platforms for sensors, ranging from satellites and aerial, to ground-based methods (**Figure 3**), achieving site-specific yield prediction across multiple spatial scales. Finally, to facilitate comprehension of readers, we have produced a nomenclature (**Table 1**).

**Table 1:** Nomenclature: abbreviations and corresponding full names.

Abbreviations	Full Name	Abbreviations	Full Name
ABA	Abscisic Acid	MCARI	Modified Chlorophyll Absorption Ratio Index
AEZ	Agro-Ecological Zone	MCYFS	Mars Crop Yield Forecasting System
AgRISTARS	Agriculture and Resources Inventory Surveys Through Aerospace Remote Sensing	ML	Machine Learning
AI	Artificial Intelligence	MLR	Multiple Linear Regression
ANFIS	Adaptive Neuro-Fuzzy Inference System	MME	Multi-Model Ensembles
ANN	Artificial Neural Network	MODIS	Moderate Resolution Imaging Spectroradiometer
APE	Agro-Pastoral Ecotone	MRE	Mean Relative Error
APSIM	Agricultural Production System Simulator Next Generation	MS	Multispectral
AVHRR	Advanced Very-High-Resolution Radiometer	MSE	Mean Squared Error
CC	Canopy Cover	MSS	Multispectral Scanner
CGM	Crop Growth Model	MTY	Marketable Tuber Yield
CGMS	Crop Growth Monitoring System	NDVI	Normalized Difference Vegetation Index
CI	Chlorophyll Index	NOAA	National Oceanic and Atmospheric Administration
CI1	Red-Edge Chlorophyll Index 1	NRCT	Normalized Relative Canopy Temperature
CI2	Red-Edge Chlorophyll Index 2	OLI	Thematic Mapper Plus
CIP	International Potato Center	PAR	Photosynthetically Active Radiation
CT	Computed Tomography	PPI	Potato Productivity Index
CV	Computer Vision	PROSPECT	Leaf Optical Properties Spectra Model
DL	Deep Learning	RF	Random Forest
EnKF	Ensemble Kalman Filter	RS	Remote Sensing
EOS	Earth Observing System	RVI	Ratio Vegetation Index
ETM+	Enhanced Thematic Mapper Plus	SAIL	Scattering By Arbitrarily Inclined Leaves Model
EVI	Enhanced Vegetation Index	SAR	Synthetic Aperture Radar
FAO	Food And Agriculture Organization	SAVI	Soil-Adjusted Vegetation Index
FPAR	Fraction Of Absorbed Photosynthetically Active Radiation	SDGs	Sustainable Development Goals
GA	Genetic Algorithm	SLR	Simple Linear Regression
GF-1	Gaofen-1	SVM	Support Vector Machine
GLUE	Generalized Likelihood Uncertainty Estimation	TCI	Temperature Condition Index
HI	Harvest Index	TIR	Thermal Infrared
HS	Hyperspectral	TM	Thematic Mapper
IIASA	International Institute for Applied Systems Analysis	UAV	Unmanned Aerial Vehicle
LACIE	Large Area Crop Inventory Experiment	UN	United Nations
LAI	Leaf Area Index	VCI	Vegetation Condition Index
LF	Limiting Factor	VI	Vegetation Index
LiDAR	Light Detection and Ranging	WDVI	Weighted Difference Vegetation Index
LSTM	Long Short-Term Memory Networks	WOFOST	World Food Studies
LUE	Light Use Efficiency	WP	Water Productivity
MARS	Monitoring Agricultural Resources	4DVAR	Four-Dimensional Variational Data Assimilation

## 3 Remote sensing for potato yield prediction

Agricultural RS was primarily applied in the resource survey, and in crop growth monitoring, yield prediction, disaster estimation, and loss assessment (Weiss et al., 2020). The LACIE program in the 1970s was representative of RS-based yield prediction in other crops; related applications in potatoes have been relatively delayed. In the 1980s, researchers employed satellite imagery to estimate potato acreage and combined it with yield data to estimate production (Ryerson et al., 1985). Nonetheless, during this period, yield data were still obtained through interviewing farmers, rather than direct evaluation of RS imagery. In 1992, potato yield was estimated by combining process-based crop models with leaf area index (LAI) data collected by handheld multispectral sensors (Finke, 1992). However, the spatial coverage of the handheld instruments was incomplete, which made it challenging to capture the yield variability of the entire field. In the early 21st century, several studies were conducted using non-destructive and convenient UAVs and satellites, which provided more comprehensive spatial coverage, for potato yield estimation (Yokobori et al., 2004; Bala and Islam, 2009). Currently, the technology for RS-based potato yield prediction has significantly advanced with the emergence of new-generation platforms, sensors, and advanced algorithms.

In this section RS-based yield prediction methods were reviewed from three perspectives: the acquisition of RS information, the selection of modeling parameters and the evolution of yield prediction models.

### 3.1 Acquisition of RS information

The RS system is comprised of a platform and integrated sensors. Different types of RS platforms offer unique benefits for specific application scenarios. According to the type of platform, we divided the potato yield prediction method based on RS into satellite-based, aerial-based, and ground-based methods for evaluation.

#### 3.1.1 Satellite-based RS

Primarily, satellites equipped with spectral sensors can obtain ground vegetation spectral information over large areas for the purposes of land resource surveying, crop growth monitoring, and yield prediction (Nakalembe et al., 2021). Since 1972, satellites such as the Landsat-1, which is equipped with a Multispectral Scanner (MSS) containing four spectral bands, have been successfully launched. As a result, humanity began monitoring global resources and environmental factors on a large scale. Landsat imagery was used to estimate potato acreage by Statistics Canada in New Brunswick from 1980 to1982. They found that potato acreage could be estimated accurately using Landsat images, with a coefficient of variation of around 5.5% (Ryerson et al., 1985). A series of weather observation satellites, such as NOAA-6 with the Advanced Very-High-Resolution Radiometer (AVHRR), has been operated by the National Oceanic and Atmospheric Administration (NOAA) since 1979. These satellites have provided an ample amount of RS imagery for accurate potato yield prediction (Akhand et al., 2016).

Even the revisit time of NOAA satellites equipped with AVHRR sensors is 12 hours, the spatial resolution is only 1.1 km. In 1999, the Earth Observing System (EOS) program launched Terra, which carries five specially designed sensors for monitoring environmental and climate change. Terra carries a Moderate Resolution Imaging Spectroradiometer (MODIS) capable of receiving spectral information of 36 bands between 0.4 and 14.4 μm with a spatial resolution of 250-1000 m. The potato yield was estimated using Normalized Difference Vegetation Index (NDVI), LAI, and Fraction of Absorbed Photosynthetically Active Radiation (FPAR) extracted from Terra-MODIS with an average error rate at 15% compared with actual yield by Bala and Islam (2009).

Despite significant enhancements in the resolution of MODIS imaging sensor compared with the NOAA satellites, the mixed image components comprising of different objects such as soil and potato canopy remain challenging to be discriminated. Low-resolution satellite imaging encounters the obstacle in estimating potato yields in relatively smaller regions. Landsat 4 and subsequent satellites equipped with Thematic Mapper (TM), Enhanced Thematic Mapper Plus (ETM+), or Operational Land Imager (OLI), featuring a spatial resolution from 30 to 120 meters. The ongoing Landsat 8 and Landsat 9 observation missions each offer a revisit cycle of around 16 days. The joint utilization of both satellites has the potential to halve the revisit period to 8 days. Similarly, the Sentinel-2 of Copernicus Programme captures images of the same region every 10 days with a high spatial resolution ranging from 10 to 60 meters. Furthermore, the successful integration of Sentinel-2A and Sentinel-2B has the capability to reduce the revisit interval to a mere 5 days.

Satellites with greater spatial resolution and shorter revisit cycles showed better potential in predicting potato yield. Al-Gaadi et al. (2016) compared the accuracy of yield estimation for potato using Landsat-8 and Sentinel-2. Landsat-8 exhibited a range of prediction errors, ranging from 7.9% to 13.5%, along with R^2^ values ranging from 0.39 to 0.65 at different sites. In contrast, Sentinel-2 demonstrated a lower prediction error, falling between 3.8% and 10.2%, though there was no significant improvement in R^2^, which was between 0.47 and 0.65.

Ultra-high-resolution satellites with meter and sub-meter spatial resolution have emerged in recent years providing high-quality RS imaging data for monitoring crop development. RS employed in satellites has recently undergone substantial development, resulting in significant advancements in spatial, spectral, and temporal resolution. Nevertheless, the current cost of high-precision images acquired by commercial satellites remains high, and it is challenging for free satellite imagery at low spatial resolution to provide high-accuracy yield prediction. Moreover, apart from resolution and cost, weather conditions like cloud cover can also limit the data quality of obtained vegetation spectra. Site-specific potato dry matter yield was estimated using GeoEye-1 with an R^2^ value of 0.60 (Elmetwalli et al., 2014). Due to the cost reduction in satellite launch, a number of commercial companies have launched small satellites that can be leveraged for Earth observation. PlanetScope, launched by Planet comprising 130 small satellites that can capture daily multispectral images at 3-meter resolution. PlanetScope images were utilized to develop potato yield prediction models in Idaho and applied them to assess yield differences between Norkotah and Russet varieties in Lebanon (Abou Ali et al., 2020). **Table 2** displays several satellites and sensors that have been utilized for potato yield prediction in recent times. Meanwhile, we present some cases of potato yield prediction with satellites in **Table 3**.

**Table 2:** Satellites that have previously been used to predict potato yields.

Platform	Sensors	Bands Number	Bands range*	Revisit interval (days)	Spatial Resolution (m/pixel)	Cost	Running state on orbit
NOAA	AVHRR	5-6	VIS, NIR, MIR, TIR	0.5	1100	Free	In progress
Landsat 5	TM	7	VIS, NIR, SWIR, TIR	16	30, 120	Free	Decommissioned in 2013
Landsat 7	ETM+	7	VIS, NIR, SWIR, TIR, PAN	16	15, 30, 60	Free	Decommissioned in 2021
Terra	MODIS	36	VIS, NIR, SWIR, MIR, TIR	1-2	250, 500, 1000	Free	In progress
IRS P6	LISS-3	4	VIS, NIR	24	23.5	Paid	In progress
GeoEye-1	–	4	VIS, NIR	3-5	0.41-1.65	Paid	In progress
Landsat 8, 9	OLI/TIRS	11	VIS, NIR, SWIR, TIR, PAN	16	15, 30, 100	Free	In progress
PlanetScope	PS2, PS2-SD	4-5	VIS, RE, NIR	1-2	3-4	Paid	In progress
Sentinel-2A/2B	MSI	13	VIS, RE, NIR, SWIR	10	10, 20, 60	Free	In progress

*Meaning of the abbreviations. MIR, Mid-Infrared; MW, Microwave; NIR, Near Infrared; PAN, Panchromatic; RE, Red edge; SWIR, Shortwave Infrared; TIR, Thermal Infrared; VIS, Visible Spectrum.

**Table 3:** Summary of satellite-based potato yield prediction.

Testing year	Country	Satellite	Sensor Type	Sensor Model	Cultivars	Parameters	Modelling Method	Accuracy	Sample size	References
2005/06-2006/07	Bangladesh	Terra	MS^1^	MODIS	–	Regional level: NDVI^2^, LAI^3^, FPAR^4^, Field level: mean NDVI	SLR^23^	Regional level: R^2^ (NDVI) = 0.79; R^2^ (LAI) = 0.87; R^2^ (FPAR) = 0.83; Field level: R^2^ (mean NDVI) = 0.66	6 (Regional) 50 (Field)	Bala and Islam, 2009
2004-2005	United States	Landsat 5	MS	TM	–	ISAVI^5^, cumulative ET^6^	MLR^24^	R^2^ (2004) = 0.97; R^2^ (2005) = 0.75	9 (2004) 13 (2005)	Sivarajan, 2011
2011	Libya	GeoEye-1	MS	–	–	NDVI	SLR	R^2^ = 0.60	24	Elmetwalli et al., 2014
1980-2014	Bangladesh	NOAA	MS	AVHRR	–	VCI^7^, TCI^8^	ANN^25^	error % < 10%	24	Akhand et al., 2016
2016	Saudi Arabia	Landsat 8, Sentinel-2A	MS	OLI/TIRS, MSI	–	NDVI, SAVI, cumulative NDVI, cumulative SAVI	SLR	R^2^ (Landsat 8) = 0.39-0.65 R^2^ (Sentinel-2A) = 0.47-0.65	60	Al-Gaadi et al., 2016
2010/11-2015/16	Bangladesh	Landsat 5, 7, 8	MS	TM, ETM+, OLI/TIRS	–	Mean NDVI	SLR	R^2^ = 0.81	6	Newton et al., 2018
2016-2018	Spain	Sentinel-2A, 2B	MS	MSI	–	ARI2^9^, CRI2^10^, IRECI2^11^, LCC^12^, NDVI, PSRI^13^, WDVI^14^, S2 bands^15^	GLM^26^, rqlasso^27^, LeapBack^28^, svmL^29^, svmR^30^, MARS^31^, kknn^32^, RF^33^, avNNet^34^	The best three algrithoms: R^2^ (rqlasso) = 0.90; R^2^ (LeapBack) = 0.89; R^2^ (svmR) = 0.93	33	Gómez et al., 2019
2012/13-2017/18	India	IRS P6	MS	LISS-3	–	VCI, climate data	Step wise regression	RMSE = 9.8-21.8%	–	Kumar et al., 2019
2017	United States, Lebanon	PlanetScope	MS	PS2, PS2-SD	Russet Burbank, Norkotah	SAVI	OLS35	R^2^ (Russet Burbank) = 0.44; R^2^ (Norkotah) = 0.57	–	Abou Ali et al., 2020
2004-2018	Mexico	Terra	MS	MODIS	–	NDVI, Harvested last year, climate, irrigation	RF, svmP, svmL, svmR, GLM	R^2^ (RF) = 0.757-0.839; R^2^ (svmR) = 0.733-0.837; R^2^ (svmL) = 0.692-0.863; R^2^ (svmP) = 0.717-0.858; R^2^ (GLM) = 0.612-0.834	838	Salvador et al., 2020
2016-2019	Spain	Sentinel-2	MS	MSI	Monalisa, Spunta, Rudolf	NDVI, PPI^16^, S2 bands	RF, SVM	S2 & PPI: R^2^ (RF) = 0.77; R^2^ (SVM) = 0.63 S2 & NDVI: R^2^ (RF) = 0.66; R^2^ (SVM) = 0.64	40	Gómez et al., 2021
2019-2020	United Kingdom	Sentinel-2	MS	MSI	Maris Piper, Amora, Pentland Dell	NDVI, SLAVI^17^, NDMI^18^, CIG^19^	SLR	R^2 = ^0.65, NRMSE = 0.16	94	Mhango et al., 2021
2020	India	Sentinel-2	MS	MSI	–	NDVI	SLR	R^2^ = 0.692	50	Singha and Swain, 2022
2016-2018	Belgium	Sentinel-2	MS	MSI	–	NDVI integral, Tmax^20^, P^21^, SDrz^22^	RF	R^2^ (Late potato) = 0.57; R^2^ (Early potato) = 0.68	723	Vannoppen and Gobin, 2022

^1^ MS, Multispectral; ^2^ NDVI, Normalized Difference Vegetation Index; ^3^ LAI, Leaf Area Index; ^4^ FPAR, Fraction of Absorbed Photosynthetically Active Radiation; ^5^ ISAVI, three-date Integrated Soil Adjusted Vegetation Index; ^6^ ET, Evapotranspiration; ^7^ VCI, Vegetation Condition Index; ^8^ TCI, Temperature Condition Index; ^9^ ARI2, Anthocyanin Reflectance Index; ^10^ CRI2, Carotenoid Reflectance Index; ^11^ IRECI2, Inverted Red-Edge Chlorophyll Index; ^12^ LCC, Leaf Chlorophyll Content; ^13^ PSRI, Plant Senescence Reflectance Index; ^14^ WDVI, Weighted Difference Vegetation Index; ^15^ S2 bands, Sentinel-2 bands; ^16^ PPI, Potato Productivity Index; ^17^ SLAVI, Specific Leaf Area Vegetation Index; ^18^ NDMI, Normalized Difference Moisture Index; ^19^ CIG, Chlorophyll Index Green; ^20^ Tmax, monthly maximum temperature; ^21^ P, monthly precipitation; ^22^ SDrz, daily root-zone soil water depletion; ^23^ SLR, Single Linear Regression; ^24^ MLR, Multiple Linear Regression; ^25^ ANN, Artificial Neural Network; ^26^ GLM, Generalised Linear Model; ^27^ rqlasso, Quantile Regression with LASSO penalty; ^28^ LeapBack, Linear Regression with Backwards Selection; ^29^ svmL, Support Vector Machine Linear; ^30^ svmR, Support Vector Machine Radial; ^31^ svmP, Support Vector Machine Polynomial; ^32^ MARS, Multivariate adaptive regression splines; ^33^ RF, Random Forest; ^34^ kknn, k-Nearest Neighbours; ^35^ avNNet, Averaged Neural Network.

For the past decades, RS employed in satellites has undergone substantial development, resulting in significant advancements in spatial, spectral and temporal resolution. Nevertheless, the current cost of high-precision images acquired by commercial satellites remains high, and it is challenging for the free satellite imagery at low spatial resolution to provide high-accuracy yield prediction. Moreover, apart from resolution and cost, weather conditions like cloud cover can also limit the data quality of obtained vegetation spectra.

#### 3.1.2 Aerial-based RS

Aerial-based RS platforms include aerial vehicles at high altitudes and UAVs at low altitudes. A fixed-wing aerial plane Piper Seneca equipped with multispectral cameras was used to capture images of southern Idaho to estimate irrigated potato yield, which can be performed as a flexible and effective tool for yield prediction (Sivarajan, 2011). However, the cost for fuel and professional pilot is high. Recently, UAVs has become an important tool for RS-based yield prediction owing to its advantages of high resolution, high throughput, and low cost (Yang G. et al., 2017). Compared with satellites and manned aircraft, UAVs equipped with high-resolution sensors are able to acquire more detailed vegetation phenotypic information to predict yield. Most of the UAVs for field phenotyping fly at an altitude of below 150 m (Stöcker et al., 2017), and the image resolution can reach the centimeter level. There are several kinds of UAVs used in agriculture, such as multi-rotor UAVs, fixed-wing UAVs and unmanned helicopters. Multi-rotor UAVs are able to hover and turn flexibly in the air (Fu et al., 2020) but with high power usage, which lead to short battery life mostly within 30 minutes. In addition, multi-rotor UAV can carry limited number and types of sensors due to the small payload. Fixed-wing UAVs can fly at high speed with longer battery life, allowing them to cover a large area of farmland in a short period of time. In addition, fixed-wing UAVs with large wings typically have larger payloads which can offer a wider sensor option. However, it is impossible for fixed-wing UAV to capture data in small-scale farms because of the long runways required for takeoff and landing, and the inability to hover and turn flexibly in the air. Multi-rotor UAV is mostly used for potato yield forecasting, which is also for current mapping operations. Although we have not yet found the application of fixed-wing drones in potato yield prediction, they have great potential for large-scale potato field monitoring due to their high speed, long endurance, and large loads.

Compared to satellites that carry a fixed number and type of sensors, UAVs can readily change to appropriate sensors to meet specific needs. For example, it is feasible to extract information such as vegetation structure and reflectance from high-resolution RGB images for growth monitoring and biomass estimation. In contrast to digital RGB cameras that can function in the visible range, multispectral (MS) cameras obtain images at multiple spectral bands, including near infrared, which provides supplemented spectral information to estimate yield by calculating vegetation indexes (VIs). With the relatively low price of RGB and MS cameras, researchers often choose affordable small or medium-sized UAVs to conduct field trials. Most MS can only acquire a small amount of spectral information with low spectral resolution in the visible and near-infrared bands. In contrast, hyperspectral (HS) cameras provide higher spectral resolution with more continuous spectral information than MS. The above-mentioned spectral sensors have specific requirements for weather conditions when performing their tasks; in particular MS and HS need to acquire images in clear and cloud-free conditions.

Relative to passive sensors, active sensors can obtain highly accurate phenotypic information, such as plant height and biomass, independent of sunlight (ten Harkel et al., 2020). Light Detection and Ranging (LiDAR) and Synthetic Aperture Radar (SAR) are typical active sensors available on the market today. LiDAR obtains 3D and echo intensity information of vegetation to monitor crop growth based on the backward scattering characteristics of the light feature (Raj et al., 2020). SAR is a high-resolution active microwave imaging detection sensor that can penetrate clouds to obtain crop phenotypic information independent of atmospheric conditions and solar radiation (Lyalin et al., 2018). Active sensors such as LiDAR and SAR have not been applied in potato yield prediction. Due to the high cost of HS imaging sensor, reliable UAVs, such as the DJI Matrice 600 Pro (DJI Technology Co., Shenzhen, China) is preferred. Despite the advantages of UAVs for yield estimation at a large scale, there are still fewer studies on the application of UAV for potato yield prediction comparing to other staple crops. For reference, we summarize some previous studies of potato yield prediction combined with UAVs in **Table 4**.

**Table 4:** Summary of aerial-based potato yield prediction.

Country	Platform Type	Platform Model	Sensor Type	Sensor Model	Cultivars	Bands number	Bands range	Parameters	Modelling Method	Accuracy	Sample size	References
United States	Plane	Piper Seneca	MS^1^	three Kodak Megaplus 4.2i digital cameras	–	3	G, R, NIR	Integrated SAVI	SLR^11^	R^2^ = 0.89	18	Sivarajan, 2011
Japan	UAV	YH300 (Yammer Ltd.)	MS	MSIS, MS2100, DuncanTech, Ltd.	Toyoshiro	4	VIS, NIR	SPAD, stem length	MLR^12^	R^2^ = 0.75	10	Yokobori et al., 2004
Japan	UAV	DJI Spreading Wings S900	MS	Micro MCA RGB+3, Tetracam	Toyoshiro	6	425-950 nm	NDVI, height	Alex Net, SLR, MLR	R^2^ (NDVI) = 0.18-0.67; R^2^ (NDVI & height) = 0.12-1.00; Alex Net: Insufficient precision	23	Tanabe et al., 2019
China	UAV	DJI Matrice 600 Pro	HS^2^	Headwall Nano-Hyperspec (Headwall Photonics Inc., Bolton, MA, USA)	Favorita; Shepody; Zhongshu 5/10/18/19	272	400-1000 nm	MCARI^3^, CI1^4^, CI2^5^, MCARI/OSAVI^6^, full spectrum, height	PLSR^13^, RF^14^	R^2^ (PLSR) = 0.81; R^2^ (RF) = 0.63	144	Li et al., 2020
United States	UAV	DJI Matrice 600 Pro	HS	Headwall Nano-Hyperspec (Headwall Photonics Inc., Bolton, MA, USA)	6 unknown varieties	273	400-1000 nm	full spectrum	Ridge^15^; OLS^16^; PLSR; SVM^17^; RF; AdaBoost	R^2^ (ridge) = 0.63; R^2^ (OLS) = 0.13; R^2^ (PLSR) = 0.53; R^2^ (SVR) = 0.57; R^2^ (RF) = 0.51; R^2^ (AdaBoost) = 0.45;	96	Sun et al., 2020
United States	UAV	DJI Phantom 4, DJI Inspire 2	MS	NIR + regular camera/senser; Altum multispectral sensor (MicaSense, Seattle, WA, USA)	Russet Burbank; Shepody; Superior	4, 6	VIS, RE, NIR, LWIR	ANTHO^7^, GNDVI^8^, BNDVI^9^; CHLGR^10^, NDVI	GLM^18^	R^2^ = 0.34-0.63	288	Jasim et al., 2020
China	UAV	DJI Matrice 600	HS	regular camera, Cubert UHD-185	Zhongshu 3/5	125	450-950 nm	–	PLSR	R^2^ = 0.74, NRMSE = 22.37%	42	Wu et al., 2020
United States	UAV	DJI Inspire 2	MS	GEMS multispectral camera (Sentek Systems LLC, Minneapolis, MN, USA)	–	4	R, G, B, NIR	cultivar information	RF, SVM	R^2^ = 0.75-0.79	–	Li et al., 2021

^1^ MS, Multispectral; ^2^ HS, Hyperspectral; ^3^ MCARI, Modified Chlorophyll Absorption Reflectance Index; ^4^ CI1, Red-edge Chlorophyll Index 1; ^5^ CI2, Red-edge Chlorophyll Index 2; ^6^ OSAVI, Optimised Soil Adjusted Vegetation Index; ^7^ ANTHO, Anthocyanin; ^8^ GNDVI, Green Normalized Difference Vegetation Index; ^9^ BNDVI, Blue Normalized Difference Vegetation Index; ^10^ CHLGR, Chlorophyll Green; ^11^ SLR, Single Linear Regression; ^12^ MLR, Multiple Linear Regression; ^13^ PLSR, Parcial Least Squares Regression; ^14^ RF, Random Forest; ^15^ Ridge, Ridge Regression; ^16^ OLS, Ordinary Least Squares; ^17^ SVM, Support Vector Machine; ^18^ GLM, Generalised Linear Model.

#### 3.1.3 Ground-based RS

Ground-based methods provide higher resolution and more angular image data for crop field phenotype. Many ground-based platforms have been developed, such as handheld or bracketed devices, ground carriers, tracks, ropeways, and fixed towers. They have their own unique advantages for different applications. Handheld or bracketed devices are simple and flexible in acquiring data. Tracks, ropeways, and fixed towers provide continuous observation of the same plot with high-precision sensors. However, they can only acquire data of specific plant samples. In contrast, ground carriers can perform data acquisition tasks over relatively larger areas.

According to our literature review, handheld or bracketed devices are still the most applied ground-based platforms for potato yield prediction. Different sensors have been used to predict potato yield by correlating various factors with yield. Zaeen et al. (2020) combined chlorophyll index (CI) and multiple VIs obtained by active sensors (Crop Circle™ and GreenSeeker™) to improve the performance of potato yield prediction. Their results indicated that the 18^th^ and 20^th^ leaf growth stages were the optimal period for data collection. No significant difference in accuracy between active spectral sensor and passive sensor was found for yield prediction in the early season by comparing handheld active sensors (Crop Circle™ and GreenSeeker™) with the portable MS Altum (MicaSense, Seattle, WA, USA) equipped on UAVs (Jasim et al., 2020). To the best of our knowledge, active sensors such as SAR and LiDAR have not been applied in potato yield prediction.

Proximal handled thermal infrared (TIR) imaging sensors could obtain canopy temperature at higher accuracy, but the accuracy decreased to ±5°C when integrating with UAV due to the impact of environmental conditions for potato yield prediction (Kelly et al., 2019). By capturing imaging data with combining a handheld infrared camera (Ti-32, Fluke Thermography, Glottertal, Germany) and a digital RGB camera (D5100 reflex, Kodak, Tokyo, Japan), an integrated adaptive neuro-fuzzy inference system with a genetic algorithm (ANFIS-GA) was used to predict yield of two potato varieties in a dry crop trial in Egypt (Elsayed et al., 2021). Similarly, we list some studies for potato yield prediction by ground-based RS in the **Table 5**. Other platforms were not identified for potato yield prediction.

**Table 5:** Summary of ground-based potato yield prediction.

Country	Platform Type	Sensor Type	Sensor Model	Cultivars	Bands number	Bands range (nm)	Parameters	Modelling Method	Accuracy	Sample size	References
Germany	Handheld	MS^1^	hand-held multi-spectral radiometer (CROPSCAN, Inc.)	–	2	670, 870	LAI^4^, model input parameters	assimilation of LAI and LEACHN model	r = 0.63	36	Finke, 1992
Canada	Handheld	HS^2^	FieldSpec HandHeld spectroradiometer (Analytical Spectral Devices [ASD] Inc., Boulder, CO)	Russet Burbank	200	325-1075	CI1^5^	SLR^13^	r = 0.64	40	Morier et al., 2015
United States	Handheld	MS	GreenSeeker™ (Trimble Navigation Limited, Sunnyvale, CA, USA); Holland Scientific Crop Circle™ ACS 430 (Holland Scientific, Inc., Lincoln, NE, USA)	Russet Burbank	GreenSeeker: 2; Crop Circle: 3	GreenSeeker: 660, 770; Crop Circle: 650, 730, 760	NDVI^6^, proprietor-proxy LAI	nonlinear regression	R^2^ (GreenSeeker) = 0.60; R^2^ (Crop Circle) = 0.64	144	Sharma et al., 2017
United States	Handheld	MS	GreenSeeker™;Holland Scientific Crop Circle™ ACS 430	Russet Burbank, Shepody, Superior	GreenSeeker: 2; Crop Circle: 3	GreenSeeker: 660, 770; Crop Circle: 650, 730, 760	NDVI; NDVI, NDRE^7^, CHLRE^8^, LAI	nonlinear regression	R^2^_adj_ (GreenSeeker) = 0.57; R^2^_adj_ (GreenSeeker) = 0.36	288	Jasim et al., 2020
China	Handheld	HS	USB 2000 spectrometer (Ocean Optics, Inc., Dunedin, Florida, United States)	Shepody	1630	350-1100	OCW-based CI	SLR	R^2^ = 0.8225; RMSE = 0.2257	27	Luo et al., 2020
Canada	Handheld	MS	FieldScout CM 1000 NDVI Meter (Spectrum Technologies, Aurora, USA)	Russet Burbank	2	660, 840	NDVI, soil data	SLR; EN^14^; k-NN^15^; SVM^16^	R^2^ (SVR) = 0.54-0.72; R^2^ (SLR) = 0.53-0.70; R^2^ (EN) = 0.49-0.64; R^2^ (k-NN) = 0.53-0.64	479	Abbas et al., 2020
Egypt	Handheld	Thermal, RGB^3^	handheld infrared thermal camera (Ti-32; Fluke Thermography, Glottertal, Germany); 14-megapixel digital camera (Kodak D5100 reflex; Tokyo, Japan)	Bellini, Arizona	–	7500-14000, 400-700	color Indices, NRTC^10^	SMLR^17^; ANFIS^18^-GA^19^	R^2^ (SMLR) = 0.73; R^2^ (ANFIS-GA) = 0.80	48	Elsayed et al., 2021
Peru	Handheld	Thermal, RGB	FLIR thermal camera (Model E60, FLIR Systems Inc., Täby, Sweden); digital camera D5300 (Nikon, Thailand)	Unica	–	7500-13000, 400-700	CC^11^, dT^12^, model input parameters	assimilation of dT and SOLANUM model	R^2^ = 0.91-0.99	8	Ninanya et al., 2021

^1^ MS, Multispectral; ^2^ HS, Hyperspectral; ^3^ RGB, red-green-blue; ^4^ LAI, Leaf Area Index; ^5^ CI1, Red-edge Chlorophyll Index 1; ^6^ NDVI, Normalized Difference Vegetation Index; ^7^ NDRE, Normalized Difference Red-edge; ^8^ CHLRE, Chlorophyll Red-edge; ^9^ OCW, Optimal Combination Weighting Method; ^10^ NRTC, Normalized Relative Canopy Temperature; ^11^ CC, Canopy Cover; ^12^ dT, Canopy temperature minus air temperature; ^13^ SLR, Single Linear Regression; ^14^ EN, Elastic Net; ^15^ k-NN, k-nearest neighbor; ^16^ SVM, Support Vector Machine; ^17^ SMLR, Stepwise Multiple Linear Regression; ^17^ ANFIS, Adaptive Neuro-fuzzy Inference System; ^19^ GA, Genetic Algorithm.

### 3.2 Selection of RS-based modeling parameters

The spectral, structural, thermal, and textural information of crop canopies are important indicators to explain yield variability (Maimaitijiang et al., 2020). Reflected light from the canopy allows us to estimate the photosynthetic capacity and other crop growth conditions of plants to predict yield. VIs highlight image spectral features to analyze crop phenotypic traits by fusing reflectance information from two or more bands; the normalized difference vegetation index (NDVI) is the most used VI in agriculture RS (Huang et al., 2021). In addition to NDVI, other VIs such as Soil-Adjusted Vegetation Index (SAVI), Ratio Vegetation Index (RVI) and Enhanced Vegetation Index (EVI) are also used in the field of potato yield prediction. Recently, Potato Productivity Index (PPI), calculated based on the two bands at 490 to 945 nm bands considering the key role of water stress on potato tuber development and yield formation, was designed for potato production practices by Gómez et al. (2021). All bands of Sentinel-2, NDVI, PPI, coupled with a random forest (RF) model were adopted to predict potato yield, with the R^2^ of 0.77. In addition to moisture, temperature is also an important environmental factor affecting potato tuber development. TIR cameras can generate thermal indices such as normalized relative canopy temperature (NRCT) to monitor temperature change on canopy to monitor drought tolerance (Elsayed et al., 2021). The difference between canopy temperature and air temperature can also reveal the water stress status of potato. The full spectral bands obtained by HS cameras provide more spectral information in visible and near infrared region for potato yield prediction than the spectral index above. Potato yield was predicted by using full-band spectra between 400 and 1000 nm (R^2^ = 0.81), which had better performance than using plant height and several VIs including Red-edge Chlorophyll Index 1 (CI1), Modified Chlorophyll Absorption Ratio Index (MCARI), Ratio2, and Red-edge Chlorophyll Index 2 (CI2) (R^2^ = 0.69) (Li et al., 2020).

It is essential to select the best period to estimate yield because of the great variation in the predicted performance of different growth periods. The weight of values at different growing periods were determined for estimating yield of potato using a handheld hyperspectral camera (Luo et al., 2020). They believed that the tuber expansion period (about 70 days after planting in this study) was the best period for potato yield prediction with an adjusted R^2^ = 0.83. In addition, it has also been noted that 90 days after planting is satisfactory for potato yield prediction (Li et al., 2020). This may be caused by the great variation in species and environmental conditions selected for different studies. Meanwhile, many studies have used time series data rather than single period data to predict yields (Fernandes et al., 2017; Aghighi et al., 2018). This could be due to the more comprehensive information on crop growth and development contained within the time-series data. For potato yield prediction, three-date Integrated SAVI (ISAVI) is a better predictor of yield than single-period SAVI (Sivarajan, 2011). Time-series data can also be applied to advanced algorithms such as Long Short-Term Memory networks (LSTM) and three-dimensional Convolutional Neural Network (3D-CNN).

Compared with spectral features, canopy traits such as LAI, plant height, and canopy cover (CC) can reflect the light use efficiency on the canopy. VIs combined with structural parameters such as plant height and LAI provides a better prediction of potato yield (Sharma et al., 2017; Tanabe et al., 2019). In contrast to passive RS, active RS comes with its own radiation source and reflects the characteristics of the ground by transmitting and receiving electromagnetic waves. Their applications are less affected by ambient light and the electromagnetic wave wavelength and emission mode can be set according to different land features, allowing them to obtain the vegetation spatial structure parameters more accurately. In addition to spectral and structural parameters, adding texture features can potentially impact the performance of yield prediction (Ma et al., 2022). However, there are no publications investigating potato yield prediction with texture features extracted from image analysis.

It is also difficult to fully represent the crop growth status by RS data alone. Integrating RS parameters with other indicators of agronomy and meteorology is an effective way to improve yield prediction capability. NDVI combined with plant height provides improved estimation accuracy of potato yield compared with using NDVI alone (Tanabe et al., 2019). Combining soil parameters, such as moisture, conductivity, and nutritional parameters, with NDVI obtained from handheld instruments, potato yield was predicted by Support Vector Machine (SVM) and the determination coefficient of different datasets ranged from 0.54 to 0.72 (Abbas et al., 2020). Likewise, RS information combined with meteorological parameters provides a good prediction of yield with the determination of coefficients ranging from 0.76 to 0.86 in winter and summer growing seasons (Salvador et al., 2020).

### 3.3 Evolution of RS-based yield prediction methods

Empirical modeling methods, such as Simple Linear Regression (SLR), Multiple Linear Regression (MLR), and Artificial Neural Network (ANN), are mostly used for current RS-based potato yield prediction. LR clearly shows the relationship between one or more explanatory variables and yield. SLR can build a linear relationship between a single parameter and the yield. Introducing more variables including VIs, agronomic parameters, and meteorology by MLR can improve model performance. However, since the relationship between the variables of the dataset is not linear in most real-life scenarios, a non-linear approach is necessary.

With the development of artificial intelligence (AI), ML models have been increasingly applied to RS-based potato yield prediction. An ANN model was constructed with variables including Vegetation Condition Index (VCI) and Temperature Condition Index (TCI) captured by NOAA-AVHRR between 1980-2014 (Akhand et al., 2016). Percentage error was calculated as lower than 10% to quantify the difference between actual and predicted yield. In addition, there are significant differences in prediction accuracy between various ML algorithms. Six ML algorithms (PLSR, Parcial Least Squares Regression; RF, Random Forest; Ridge, Ridge Regression; OLS, Ordinary Least Squares; SVM, Support Vector Machine; GLM: Generalised Linear Model) were compared for potato yield prediction at different irrigation levels using a Headwall nano-hyperspec imager, and ridge regression showed the highest accuracy with R^2^ of 0.63 (Sun et al., 2020). Yield prediction performance of several ML algorithms were compared using Sentinel-2 images and svmRadial got the highest accuracy (R^2^ = 0.93) (Gómez et al., 2019). It is also worth noting that varietal differences can significantly impact predicted results. ML combined with cultivar information and UAV-based images was used to improve potato yield prediction (Li et al., 2021). The results showed that RF and SVM models using only RS data yield poor estimation (R^2^ = 0.48-0.51) but had significantly improved performance (R^2^ = 0.75-0.79) when variety information was included. The use of ML algorithms combined with high spatial resolution images and cultivar information can significantly improve yield prediction for different potato varieties than approaches without variety information.

The AlexNet algorithm, proposed in 2012 as the first deep learning (DL) model, generates both low- and high-level features of data through a multilayer neural network as the input of fully connected layers before a classification task (Krizhevsky et al., 2017). Compared to conventional ML with handcrafted features, which reaches a bottleneck in model performance with increasing the size of training dataset, DL can further improve model performance by enlarging the training dataset due to the huge amount of generated features. The performance of MLR and AlexNet to assess potato yield was compared and concluded that the DL algorithm was superior (Tanabe et al., 2019). However, the accuracy of the proposed model was still not high enough to meet the requirement in practice, which encouraged the investigation of more complex DL networks. With the rapid development of DL, other networks including LSTM have been widely applied in yield prediction in recent years (Muruganantham et al., 2022). Many DL-based studies have been conducted in other crops (Tian et al., 2021; Liu et al., 2022). However, there is still a lack of application of such DL methods applied to potato yield prediction.

Different from the empirical models that do not have a complete mechanism of crop development, the physical model of RS considering spectra, radiation, and scattering, are deterministic based on the laws of physics. The PROSAIL, a combination of PROSPECT (leaf optical PROperties SPECTra model) and SAIL (Scattering by Arbitrarily Inclined Leaves model), considering leaf angle, canopy structure and biochemical properties of vegetation is widely used for to estimate chlorophyll content, LAI and biomass (Berger et al., 2018). A mechanistic physical model, such as the radiative transfer model, requires a thorough understanding of vegetation structure characteristics and radiative transfer theory. Probably due to high complexity of the models, physical models have not yet been applied to potato yield prediction.

Compared with the mechanistic models based on RS, the semi-empirical models allow a compromise by estimating intermediate variables or simplifying the model. The commonly used semi-empirical approach is the light use efficiency (LUE) model, which calculates dry matter yield by estimating total primary productivity and combining it with harvest index (Monteith, 1972). According to the principle of assimilates accumulation and distribution, yield could be calculated as the product of photosynthetically active radiation (PAR), fraction of absorbed PAR (FPAR), LUE, and harvest index (HI). Calculation formula is shown below:


Yield=PAR×FPAR×LUE×HI

## 4 Crop growth model for potato yield prediction

CGM delineate crop growth and development as a function of environmental factors, such as climatic, soil, and management parameters, predicated upon the physiological and ecological tenets of crops (Raymundo et al., 2014). The mechanistic simulation of potato development is an efficacious tool for predicting potato yield.

### 4.1 Evolution of potato CGM

The potato growth model has evolved from establishing fundamental principles to widespread application and continuous optimization. The original CGM was established by de Wit at Wageningen University during the 1960s (Bouman et al., 1996). Development of potato CGM began in the late 1970s when researchers designed a model based on physiological characteristics and field experiments. During this stage, the model simulated the accumulation and distribution of assimilates through potential light and thermal conditions, thereby simulating the process of potato yield formation. A simulation of potato growth was conducted by utilizing temperature, photoperiod, and soil moisture during specific time intervals (Sands et al., 1979). Light interception calculated from canopy cover was used to estimate potential potato yield (Van der Zaag, 1984). Similarly, it was posited that fundamental data such as the time of sowing and harvest, soil and air temperature, and solar radiation can be used to estimate the maximum dry matter yield of potatoes (Marshall et al., 1984). The POTATO model, crafted by Ng and Loomis (1984), is the pioneer mechanistic model for comprehensively delineating the morphology and physiology of potato. During this period, models primarily focused on productivity simulations without considering the effects of environmental stress on actual yield.

In the 1990s, soil water and nitrogen dynamic modules were successively incorporated into potato CGM. For instance, SUBSTOR-potato (Griffin et al., 1993), a sub-module of DSSAT, is utilized to simulate potato growth, with its water and nitrogen dynamics module derived from CERES (Crop Environment Research Synthesis). During the same period, numerous studies explored the optimization of potato irrigation and fertilization management schemes using CGM. Additionally, some research employed models to assess the impact of climate change on potato production.

Currently, models are being widely calibrated and validated across different regions to suit the needs of potato growth simulation. Additionally, CO_2_ response modules have been added to the CGM (Wolf, 2002), which have been extensively used for decision support and climate change response studies (Raymundo et al., 2018; Tang et al., 2021). The uncertainty in potato models caused by model structure, model input, and model parameters has already attracted the attention of researchers (Ojeda et al., 2020). This has been extensively studied in other crops (Bert et al., 2007; Wang et al., 2020). In addition, CGM utilize data of specific samples, which cannot reflect the spatial heterogeneity of large-scale farmland. Combination of the high-throughput and full-coverage advantages of RS with the complete mechanism of CGM makes the assimilation of RS and CGM an effective way to achieve continuous spatiotemporal monitoring of potato growth dynamics. For instance, in recent years, AquaCrop has emerged to simulate crop yield using CC as an intermediate variable, which is closely related to RS. However, there is still limited research on predicting potato yield using assimilation techniques. **Table 6** illustrates the application of CGM for predicting potato yield in recent years.

**Table 6:** Summary of CGM-based potato yield prediction.

Model	Country	Testing time	Varieties	Sites	Accuracy	Sample size	References
APSIM-Potato	Australia	2012/13	Russet Burbank, Moonlight	2	NRMSE = 15.4%	–	Borus et al., 2018
APSIM-Potato	China	1986-2020	–	1	R^2^ = 0.92, NRMSE = 14.48%	10	Luo et al., 2022
Aquacrop	Argentina	2009-2010	Spunta	1	R^2^ = 0.56	25	Casa et al., 2013
AquaCrop	Denmark	2013-2015	Folva	1	R^2^ = 0.63-0.98, NRMSE = 7.3-21%	–	Razzaghi et al., 2017
AquaCrop	Ethiopia	2012	Jalene	2	E = 0.84-0.96, NRMSE=3.49-8%	–	Yibrah et al., 2015
AquaCrop	Iran	2010	Kuzima	1	R^2^ = 0.9, NRMSE = 9.21%	–	Afshar et al., 2014
CropSystVB-CSPotato	United States	2001-2002	Ranger Russet	1	–	–	Alva, 2010
Daisy	Poland	2000-2006	Triada	1	RRMSE = 15.4%	–	Mazurczyk et al., 2007
Hamer-model	United Kingdom	3-15 years	–	5	average error % = 15.8	115	Ejieji and Gowing, 2000
Infocrop-potato	India	8-18 years	–	2	NRMSE = 8-10%	–	Govindakrishnan et al., 2007
LINTUL-NPOTATO	Netherlands	1996-1999	Eersteling, Bintje, Junior, Agria	2	R^2^ = 0.865, RMSE = 1.08 Mg ha^-1^	18	Van Delden et al., 2003
LINTUL-POTATO-DSS	South Africa	2013-2014	Innovator	10	R^2^ = 0.939, 0.635	6, 9	Machakaire et al., 2016
LPOTCO	Ireland, Germany, Sweden, Finland, UK, Belgium, Italy	1998-1999	Bintje	20	R^2^ = 0.65	–	Wolf, 2002
MacKerron and Waister (1985) model, Versteeg and Van Keulen (1986) model	India	1992/1993	Kufri Chandermukhi	1	error % = 4.1-25.7%	2	Prihar et al., 1995
MoDrY	Poland	1971-1996 (excluding 1984)	–	1	R^2^ = 0.64, MRE = 12.40%	25	Zyromski et al., 2013
Sands-model	Australia	–	Exton, Sebago, Kennebec, Delaware, Sequoia	9	–	–	Hackett et al., 1979
SSM-iCrop2	Iran	2017-2018	Sante, Arinda, Agria, Marfona	4	r = 0.80, RMSE = 543 g m^−2^	20	Dadrasi et al., 2020
SUBSTOR-Potato	Argentina	1979-1983, 1987-1991	Huinkul, Kennebec, Mailen and Spunta	4	R^2^ = 0.915	24	Travasso et al., 1996
SUBSTOR-Potato	Canada	1992-1993	Kennebec	8, 12	error % = 4-15%	–	Mahdian and Gallichand, 1997
SUBSTOR-Potato	Czech	1994-2002	Rosara	1	R^2^ = 0.97 (4 years)	4	Šťastná et al., 2010
SUBSTOR-Potato	Uganda, Burundi, Peru, India, USA	1980, 2002-2010	Asante, Amarilis, Kufri Bahar, Kathadin	5	RRMSE = 28.1%	26	Kleinwechter et al., 2016
SUBSTOR-Potato, AquaCrop	China	2018-2019	Zihuabai	1	DM: R^2^ = 0.37-0.68; FM: R^2^ = 0.37-0.72	12	Wang et al., 2023

### 4.2 Representative CGM

After more than 40 years of evolution, dozens of potato growth models have been built. The principles of CGM have certain commonalities. Most models include basic crop growth, meteorology, soil, and management modules. The models also have their own focuses and have formed their own schools in various parts of the world and in different application fields. In this subsection, we will introduce some common potato CGM and systematically evaluate their applications over decades.

The potential productivity of a crop can be derived by simulating the net photosynthesis and the percentage of assimilates apportioned to the tubers (Weir et al., 1984). Multiple CGM employ this underlying principle while integrating environmental modules such as soil and climate to simulate yield. Among the early potato models, POTATO stands out as a light-driven model, which completely simulates the growth and development of the crop. Nonetheless, this model is still an oversimplified representation of the crop’s growth. Potato yield was effectively modeled by modifying the POTATO model through adjusting the photosynthetic capacity of potatoes on cloudy days (Ewing et al., 1990). In contrast to POTATO, which results in overestimated yields, NPOTATO offers more accurate yield simulation (Wolf, 2002).

SUBSTOR-Potato, a light-driven model derived from CERES, is a more comprehensive and widely used model. For improved accuracy, SUBSTOR-Potato 2.0 added water and nitrogen simulation modules (Griffin et al., 1993). Over decades of research and experimentation, researchers have identified some shortcomings in the model. As the number of studies increases, the model is constantly being refined. In a Canadian study, SUBSTOR-Potato was applied to simulate yield, but an underestimation of 15% occurred due to incorrectly simulated soil moisture content (Mahdian and Gallichand, 1997). Similarly, the model predictions may still underestimate yields under extreme weather conditions. Data from 87 field experiments was synthesized and it was proposed that it is necessary to improve SUBSTOR-Potato to capture the effects of increased atmospheric CO_2_ concentration and temperature rise on crop growth (Raymundo et al., 2017). In DSSAT version 4.7, this problem was solved by modifying the response function (Raymundo et al., 2018). However, this version of DSSAT still neglects the impacts of pests and diseases on yield loss caused by quality degradation (Tooley et al., 2021).

LINTUL-Potato, which is based on the light interception and utilization model, carefully considers the influence of temperature and daylength on potato yield formation (Kooman and Haverkort, 1995). Temperature is significant in seedling emergence, light energy utilization, canopy morphogenesis, tuber bulking, and yield formation, while photoperiod has a considerable effect on light energy utilization and potato tuberization (Snyder and Ewing, 1989). By assessing the effect of freezing on yield, the simulated result of LINTUL-Potato showed that an increase in the cold tolerance of potatoes from -1°C to -2°C and -3°C led to respective increases in average yield of 26% and 40% (Hijmans et al., 2003). In addition, numerous models have been derived from LINTUL-Potato that are tailored to various scenarios. Van Delden et al. (2003) simulated nitrogen dynamics and potato yield under different organic nitrogen management strategies in the Netherlands using LINTUL-NPOTATO. Similarly, the LINTUL-Potato model was optimized for simulating yield of potatoes with different genotypes in the Andes Mountains by the International Potato Center (CIP). The revised model known as SOLANUM (Condori et al., 2010) showed acceptable results (R^2^>0.88). LINTUL-POTATO-DSS is an enhanced version of LINTUL-Potato that reduces the potential for errors during computation by utilizing fewer parameters (Haverkort et al., 2015).

Simulating the formation and distribution of photosynthetic assimilation products is essential in the CGM. Moreover, moisture dynamics are critical in determining potato yield. Some models utilize the transpiration or evapotranspiration of the crop or soil as a driver to simulate crop growth and yield formation processes. These water-driven models can assist in the development of rational irrigation practices for efficient utilization of limited water resources. The AquaCrop model (Steduto et al., 2009), developed by the Food and Agriculture Organization (FAO) of the UN, is an example of a water-driven model that calculates biomass as the product of water productivity (WP) and cumulative evapotranspiration, multiplied by a harvest index to determine yield:


Yield=B×HI=WP×∑Tr×HI

where Y is final crop yield (kg·m^-2^), B is biomass (kg·m^-2^), HI is harvest index (%), Tr is transpiration (mm), and WP is water production efficiency (kg·m^-2^·mm^-1^).

Compared to other models that require numerous input parameters, the AquaCrop model is relatively simple and demands fewer input parameters. Furthermore, AquaCrop employs CC rather than LAI to depict the canopy structure, which allows for direct use of RS data with this model (Steduto et al., 2009; Sun et al., 2017). AquaCrop was employed to achieve better simulation results for potato tuber yield under varying irrigation conditions (R^2^ = 0.98, NRMSE=0.046) (Razzaghi et al., 2017). However, it should be noted that AquaCrop demonstrated limited efficacy in simulating each indicator at higher or lower irrigation levels (Jin et al., 2019).

Another class of water-driven model integrates a CGM with a hydrological model to describe the impacts of alterations in crop water management on potato respiration and yield. A combined SWAP-WOFOST model was employed to evaluate productivity and recommended the inclusion of capillary rise and recirculation in the model to enhance the precision of potato yield prediction (Kroes et al., 2018).

The improvement of crop yields is heavily reliant on the use of fertilizers, particularly nitrogen fertilizers. Nonetheless, excessive nitrogen application can inflate production costs, harm the environment, and pose risks to human health (Zhang et al., 2015). Precise management of nitrogen fertilizer can diminish pollution while also reducing expenses. Researchers recognized the significance of accurate nitrogen management several decades ago and integrated a nitrogen simulation module into the potato model. DAISY, a one-dimensional soil-plant-atmosphere system model, can simulate crop production, soil water balance, carbon and nitrogen cycles, and so on (Plauborg et al., 2022). DAISY was employed to simulate root abscisic acid (ABA) synthesis, stomatal conductance, transpiration and yield under water-saving irrigation conditions in potato crops (Plauborg et al., 2010). Potato yield under different split-N fertigation regimes was simulated and it was observed that prolonged N fertigation consistently increased yield (Zhou et al., 2018).

The APSIM-Potato model is part of the Agricultural Production System Simulator Next Generation (APSIM) family. APSIM simulates potato development and yield formation based on radiation, temperature, photoperiod, soil water, and nitrogen balance in daily increments (Keating et al., 2003). Many studies conducted in Australia and China have focused on water, nitrogen, sowing management, and strategies for coping with climate change (Tang et al., 2021; Li et al., 2022). APSIM-Potato, however, requires additional parameters to improve model performance (Borus et al., 2018).

The World Food Studies (WOFOST) model, developed based on the SUCROS model from Wageningen University, incorporates water and soil simulation modules to primarily simulate regional-scale crop growth and yield changes (Van Diepen et al., 1989). A study that simulated the yield of early potatoes under water-limited conditions indicated that simulation results of WOFOST are sensitive to water deficits (Kulig et al., 2020). Subsequent versions of WOFOST have included a CO_2_ response module to better simulate the effects of climate change on potato yields.

### 4.3 Assimilation methods

The utilization of RS technology enables high-throughput and non-destructive acquisition of crop phenotype data in the field. However, it falls short in simulating the crop yield formation process and lacks a strong mechanistic foundation. Mechanistic CGM simulate crop growth and development as well as yield formation processes, but they use specific samples, an aspect that is lacking in spatial expansion. The assimilation of RS and CGM can leverage the advantages of both to enhance the prediction accuracy of various crop canopy state variables and yields at regional and national scales. Despite several studies being conducted on RS and CGM assimilation for other crops, limited research has been conducted in potato. LAI acquired by Gaofen-1 (GF-1) satellite data was employed as the assimilated variable coupled with DSSAT-SUBSTOR with the SCE-UA optimization algorithm for regional potato yield prediction (Duan, 2019). The mean relative error (MRE) was only 6.17%, 9.45% lower than that of unassimilated RS data. Quiroz et al. (2017) estimated single-point potato yield using CC and the weighted difference vegetation index (WDVI) corrected crop growth model SOLANUM. Current data assimilation algorithms such as Ensemble Kalman Filter (EnKF) and Four-Dimensional Variational Data Assimilation (4DVAR), have emerged, which could lead to further research progress in assimilation studies of RS and CGM. Due to the lack of application of assimilation methods, the technical gaps might increase between yield prediction of potato and other crops.

## 5 Methods based on yield limiting factor

In the past, agronomists predicted potato yields by the “visual method” using basic conditions of local agricultural production and the growth of potatoes. Considering the impact of yield-enhancing technical measures and the yearly climate on yields, they assessed potato yields per unit area visually and by experience. However, this method relies on the investigators’ experience with crop growth and development patterns and yield formation rules, which is highly subjective. When new situations arise, such as the adoption of new technologies, the promotion of superior varieties, or when crops suffer severe losses due to abnormal disasters, the judgment of crop growth status often exceeds the investigators’ experience. This method thus often results in large errors in yield estimates and is rarely used in current agricultural production. Instead, quantitative yield estimation models based on LFs have become an important method for predicting potato yields.

### 5.1 Agronomic parameters-based methods

Methods employing traditional agronomic parameters were applied in potato yield prediction before the advent of RS and CGM. Earlier studies destructively sampled tubers to record potato weight and number of potatoes to estimate yield directly (Dyke and Avis, 1953). In addition, canopy parameters could be linked to yield by reflecting the growth status. The first category is structure indicators that can directly reflect photosynthetic capacity, such as LAI and leaf number. At the canopy structure level, planting density and number of leaves were used as yield indicators and achieved a reduction of mean squared error (MSE) of 9% (Singh et al., 2020). In addition, some physiological and biochemical indicators have been adopted. The highest correlation between yield and chlorophyll content (expressed as SPAD) has been revealed with an R^2^ value of 0.663 (Meng et al., 2021). The nutritional status of the 4th leaf, as measured by the Mg DRIS index (Mgi) and N DRIS index (Ni) during the onset of tuberization, have demonstrated potential as yield predictors. Moreover, N content in stems has shown a strong correlation with marketable tuber yield (MTY), while the Ca:N ratio in stems has displayed the highest correlation with MTY.

Traditional regression methods often have inadequate simulation performance, while some innovative methods provide better prediction results. For instance, a Canadian study demonstrated that using a three-input multiple-layer perceptron (MLP) network with cumulative LAI, maximum LAI, and cumulative rainfall achieved a higher accuracy in yield estimation than MLR and SUBSTOR (Fortin et al., 2011).

Methods based on agronomic parameters often require tedious field sampling. In addition to the plant itself, environmental and management factors, among others, can affect yield. Therefore, the ability of a method to simulate different cultivation and management conditions varies.

### 5.2 Environmental parameters-based methods

Environmental factors affecting crop growth, such as meteorology, soil, pests, and diseases, could be considered yield indicators. The concept of using meteorological data to replace destructive sampling for predicting potato yield was proposed in a report in 1929 by an unknown author in the American Potato Journal. In addition, the agro-ecological zone (AEZ) model, jointly developed by the FAO of the UN and the International Institute for Applied Systems Analysis (IIASA), predicted the yield potential of different potato farming areas based on statistical data in China from 1961 to 1997 (Cai et al., 2006). In a study examining the relationship between meteorology and yield variability over multiple years, models were constructed with 35 years of data based on MLR, stepwise regression, and BP neural networks, with MREs of 6.715%, 7.811%, and 4.479% (Yang S. et al., 2017). Piekutowska et al. (2021) constructed MLR and ANN models using yield data and meteorological and management data from 2010 to 2017, with the ANN model estimating potato yield more accurately (R^2^ = 0.86).

Furthermore, soil indicators have been utilized as predictors of potato yield in some studies. For instance, ANN and MLR models were constructed that incorporated soil infiltration resistance, organic matter, microbial load, and tillage system, resulting in superior prediction of potato yield, with R^2^ values of 0.951 and 0.894, respectively (Abrougui et al., 2019). Yield was simulated based on soil apparent conductivity and achieved an R^2^ between 0.57 and 0.66 (Frąckowiak et al., 2020). However, yield loss due to pests and diseases also constitutes an essential component of yield prediction theory. It was reported that the rate of yield loss caused by 64 potato cyst nematode eggs per gram of soil ranged from 8.5% to 56% and 9% to 58%, for two experimental sites (Hajihassani et al., 2013).

Several environmental indicators can be used as yield indicators because their variability greatly affects potato yield. However, as with methods based on agronomic parameters, these methods do not reflect the full range of potato yield formation.

### 5.3 Input-output model

The approach based on input-output theory considers various agricultural inputs, including human labor, energy, fertilizer, irrigation, pesticides, and so on (Chen and Wang, 2010). Some of the input-output model for yield prediction were developed by quantifying the effects of different energy inputs on yield by combining economic mathematical models such as the Cobb-Douglas function. Potato production was estimated in Iran using input-output theory combined with ANN and ANFIS, with correlation coefficients of 0.925 and 0.987, respectively (Khoshnevisan et al., 2014). Farm potato production was compared in Iran combined with various inputs, such as manpower, machinery, diesel, fertilizer, farmyard manure, pesticides, electricity, irrigation water, and seeds (Hamedani et al., 2015). In addition, empirical statistical models consider the yield of previous years as a crucial indicator for yield estimation.

This type of model generally employs a questionnaire to acquire the different forms of energy inputs. However, the most significant aspect of questionnaire approach might be the representativeness of the respondents and the authenticity of the survey data. Additionally, there are some differences in total energy consumption and potato yield among various production models (Al-Hamed and Wahby, 2016). For instance, the energy ratio, energy productivity, and net energy of large-scale farms (>3 ha) were considerably higher than those of smaller farms (Khoshnevisan et al., 2014). These factors constrain the applicability of prediction methods at different scales.

## 6 Discussion

This study revealed the research progress of estimating potato yield since 1953. A systematic review of different methods provides an important reference for understanding potato yield prediction applications. In this section, application scenarios, and advantages and disadvantages of various methods are systematically discussed. Additionally, the trend of potato yield prediction is prospected.

### 6.1 Application scenarios for yield prediction

Different application scenarios necessitate the use of matching yield prediction methods. Yield prediction could be used to create maps of potato yield layout in various regions and to identify the most suitable planting areas within the planting structure layout. Precise pre-harvest yield prediction at the international trade level is beneficial for early adjustment of trade policies to manage evolving international trade dynamics. Furthermore, in large-scale potato production, pre-harvest yield estimation enables development of timely marketing and storage plans to ensure economic benefits. We present several application scenarios and discuss the applicable yield prediction methods below.

#### 6.1.1 Optimal allocation of resources

Potato yield forecasts at the regional and national level can support decision-making in planning growing areas and international trade. Satellite RS is the most intuitive and effective method to meet these demands because it can monitor the extent of potato cultivation over large areas while obtaining various parameters for assessing vegetation growth and predicting final yields. In contrast to traditional statistical survey methods, satellite RS can provide accurate and real-time maps of potato yield distribution. Combining yield mapping for other crops and yield projections for different geographic spaces under future climate change conditions can identify more suitable planting areas for each crop. The integration of satellite RS technology and CGM can facilitate the prediction of crop yields over large areas with greater accuracy and precision. Satellite-based RS could predict yield about two months before harvest to allow earlier development of trade strategies and ensure economic development and food security.

From a farm perspective, potato yield prediction provides decision support for marketing and storage strategies. High-resolution satellites could be used to predict potato yield for large plantings. However, it is preferable to use UAVs to acquire high-resolution image data for slightly smaller farmlands. Companies in smart agriculture could provide these special services. In addition, long-term yield forecasting services, such as MARS Crop Yield Forecasting System (MCYFS), are necessary for all stakeholders.

#### 6.1.2 Precision management

Estimating potential yield through crop models and simulating potato productivity under different environmental conditions can offer suggestions to potato farmers to reduce yield differentials, such as implementing better management strategies for irrigation, fertilization, and sowing period (Deguchi et al., 2016; Li et al., 2022). UAV RS provides a fast and non-destructive way to access field phenotypes and is an important tool for decision support in field management. Various yield estimation models can also be integrated with intelligent management systems to achieve accurate field management.

The image processing and model building for UAV remote sensing and CGM can be challenging and require specialized skills. However, many growers may not have the necessary professional background to conduct yield prediction using these methods. To address this issue, smart agriculture companies with expertise can provide data collection, analysis, and integrated delivery services. For instance, a user interface based on the input-output model was developed using C-sharp that enables direct potato yield prediction (Al-Hamed and Wahby, 2016). In addition, the CGM could provide decision support for precision management by simulating the potato growth process under different management and climate conditions. However, it is still necessary to use different varieties to calibrate CGM in different regions. Due to the complexity of CGM, some simplified models, such as AquaCrop, might be suitable.

As smartphones become more prevalent, one approach to site-specific yield forecasting is to deliver dependable yield projections and decision-making support by incorporating multiple sources of big data into mobile devices and designing an intuitive user interface for potato farmers. It is critical that the yield estimation technique is practical and comprehensible. It should provide unambiguous information as accurately as possible while remaining applicable to commercial farming practices. Dispelling user misunderstandings is crucial in facilitating replication. Conducting pilot studies and demonstrating the effects of yield estimation in specific regions is an effective means of promotion.

#### 6.1.3 Responding to climate change

Assessing the impact of climate change on potato growth and yield is beneficial for selecting the most suitable varieties and management strategies under climate change conditions. However, the impact of climate change on potato production varies due to differences in cultivation areas, growing seasons, and cultivation management practices (Bender and Sentelhas, 2020; Yagiz et al., 2020).

There are many studies pointing to a possible decline in potato yields under future climate change conditions. Applying future climate change scenarios to current potato cropping systems using an improved SUBSTOR-potato, it was pointed to a small decline in global tuber production by 2055 (-2% to -6%) and a large decline by 2085 (-2% to -26%)(Raymundo et al., 2018). DSSAT was used to simulate the yields of barley and potato under future climate change conditions (Holden et al., 2003). Non-irrigated potato tuber production in Ireland is projected to decline in 2055 and 2075 due to water shortages. A study of yield changes under future climate change scenarios for a variety of crops in Mexico using AquaCrop suggests that Mexican potato yields will decline (Arce Romero et al., 2020).

Interestingly, other studies have produced the opposite result. The possible negative effects of increased temperatures and reduced water availability for potato are offset by the positive effects of increased CO_2_ levels on water use efficiency and crop productivity (Haverkort et al., 2013). By mid-century, potato yield variability and productivity will increase in Belgium with greater variability between climate change models than spatial variability (Vanuytrecht et al., 2016). The differences in prediction results could be attributed to the different CGM and study areas used in these investigations. In addition, different climate change scenarios also produce different simulation results (Pushpalatha et al., 2021). Despite extensive research on the impact of climate change on potato yields, validation of previous climate change impact assessment results on yields is still lacking.

According to the prediction results, it is possible to implement corresponding countermeasures, such as changing the planting area or management practices. For example, optimizing sowing and irrigation strategies can improve agro-pastoral ecotone (APE) potato yield and water productivity (Tang et al., 2018). Delayed sowing and selection of medium-maturing potato varieties are important ways to cope with warm and dry climates in the APE of northern China (Li et al., 2019). WOFOST has been integrated into the Crop Growth Monitoring System (CGMS) to assess the impacts of climate change. The impact of climate change on potential productivity of potato was studied in West Bengal using the WOFOST crop growth simulation model (Dua and Sharma, 2017). They minimized the impact of climate change by selecting appropriate varieties and changing planting dates to design management strategies.

### 6.2 Comparison of different prediction strategies for predicting potato yield

Various prediction methods have their own characteristics. In this section, yield prediction strategies are compared and the knowledge gaps between potato and other crops are presented.

#### 6.2.1 RS-based methods

RS-based methods could replace other destructive sampling methods by obtaining crop phenotype through non-destructive methods to build yield prediction models. Morier et al. (2015) inferred nitrogen stress and estimated potato yield combined with a handheld hyperspectral sensor (FieldSpec handheld spectroradiometer), which obtained similar results to destructive methods. In addition, there are significant variations between different platforms and sensors for predicting potato yield.

Satellite RS can be used to monitor large areas of field with its wide coverage. However, the constraints of cloud cover and revisit cycles might limit the availability of images. Additionally, the low spatial resolution of satellite results in a blended image element composed of soil and vegetation is challenging to process. Yield prediction using satellites faces significant challenges in many regions due to small plot sizes, mixed cropping, and intercropping. Advanced satellites and image processing methods might solve these problems. Satellites used for potato yield estimation are detailed in **Table 2**. Currently, satellites equipped with spectral sensors are still prevalent, and there is a lack of active RS satellites, such as the C-SAR-equipped GF-3 with high resolution of 1 m, applied to related research. In addition, hyperspectral satellites have not been applied to predict potato yield.

Compared to satellites, proximal RS is more suitable for site-specific rather than regional yield prediction. UAVs are a promising solution for precision agriculture management, given their high resolution, low cost, and flexibility. According to our literature survey, studies on potato estimation with UAV RS have employed a variety of sensors, but there are few applications of UAV-based active sensors. Fixed-wing drones are also applied less in potato yield prediction, although their applications in more rapid monitoring of large areas of farms cannot be ignored. Ground-based RS can capture phenotypes with higher resolution. However, handheld instruments have incomplete spatial coverage and are time-consuming for sampling. Additionally, fixed ground-based phenotyping devices have limited coverage and come with high costs.

Potato yield formation can be described as the production of photosynthetic assimilates multiply by the harvest index (Khan, 2012). The growth dynamics of the aboveground canopy can directly reflect canopy light energy interception and thus affect the formation of photosynthetic assimilation products. Therefore, many practical applications in the studies we investigated have been conducted to predict potato yield by directly obtaining aboveground phenotypes from passive optical sensors. However, they can only obtain information from the top of the canopy and are susceptible to clouds and light. Active sensors that used controlled sources of radiation, such as SAR and LiDAR, are not affected by weather and clouds. Another method is to detect the underground tubers directly by sensors with penetrating ability. Longer wavelengths are more penetrating because the object absorbs less of the wave. There has been a study conducted on the use of sound waves for detecting sweet potatoes in sandy soil (Iwase et al., 2015). However, the research is still in its preliminary stages. Computed tomography (CT) can be used to obtain potato tuber phenotypes non-destructively using X-ray to penetrate the soil for precise yield prediction. Nevertheless, CT is mostly used in indoor pot experiments (Ferreira et al., 2010), and it is difficult to apply it in open conditions due to the associated radiation dangers and the requirements of a receiver after penetrating the tuber. Ground penetrating radar (GPR) is an effective means of non-destructive detection of underground targets. Although GPR is still at the research phase in the detection of underground tubers in potato with limited throughput, this type of tool has great potential for future potato yield prediction (Cheng et al., 2022). Currently, there are few studies comparing active and passive sensors for potato yield prediction, and more research is needed to evaluate the predictive performance of both.

There are some differences in potato RS-based yield estimation compared to other crops. For example, computer vision (CV) is widely used in intelligent yield prediction to identify and count fruits for further harvesting and marketing decisions (Ramu and Priyadarsini, 2021), but potato tubers located underground are difficult to observe directly by conventional optical sensors. Sound waves and CT could obtain tuber phenotype without the effect of soil, but most studies are limited to potted plants. In complex field conditions, there is a lack of research on obtaining potato tuber phenotype directly for predicting yield. Furthermore, the leaves of potatoes are compound, and the propagation path of light between the leaves is different from that of rice and other field crops. It is necessary to improve or establish a dedicated RS-based yield prediction methods for potatoes, taking into account the inherent characteristics of potato.

Compared to the mechanistic model, most RS-based methods build empirical models by observing aboveground phenotype directly. In addition, these complex relationships between crop growth and external environmental factors, including meteorological conditions, soil nutrients, and field management, remain difficult to accurately capture through RS.

Despite significant progress in the development of theoretical models and operational systems for RS yield estimation, the limitations of current RS technology prevent accurate and quantitative reflection of the underlying mechanisms of crop development and yield formation.

#### 6.2.2 CGM-based methods

CGM simulate yield with comprehensive information of variety, management, meteorological parameters, and soil. CGM thus have a complete mechanism to simulate the crop growth process, which is their most significant property. In addition, along with yield prediction, CGM can simulate other elements such as water and nitrogen dynamics of crops to provide support for precision management. Finally, combining CGM with climate change models could predict the yield potential under different regions, cultivars, and cultivation strategies under future climate change conditions to make optimal decisions.

However, most CGM make accurate simulations based on numerous input parameters, which is often difficult to achieve in potato production. In addition, CGM require laborious field trials to calibrate and validate the models for different varieties in different regions. Somewhat simplified CGM, such as AquaCrop and LINTUL-POTATO-DSS, which significantly reduce the number of input parameters, have also been developed in recent years. Additionally, the establishment of a database platform of shared variety parameters helps to enhance the applicability of the models.

Compared to large scale RS methods, CGM cannot reflect the variation of yield in different spaces by collecting data from specific samples. Assimilation of RS with CGM could extend the model application to a regional scale. In addition, although some models use a constant HI, the effectiveness of such an approach is not always satisfactory. Techniques for assimilating external observations into the model to continually adjust certain state variables and attributes can be used to enhance model performance. RS data can offer prompt updates on crop or environmental conditions, allowing for periodic updates of model simulations during the simulation process (Hao et al., 2021).

Currently, DSSAT-SUBSTOR is still the most widely used model for predicting potato yield. However, due to differences in the modeling principles of individual models, these models often exhibit different simulation results. Uncertainty due to differences in model structure could be reduced by multi-model ensembles (MME), which could improve the prediction performance compared to a single model (Martre et al., 2015).

In addition to yield, commercial attributes of potato are also very important. Tuber yield and tuber size (expressed as number of tubers per 10 kg) were simulated using LINTUL-POTATO-DSS (Machakaire et al., 2016). However, there were few studies that simulated commercial indicators such as potato tuber size. Additionally, unpredictable extreme weather events remain a concern, and even with the ability to anticipate and forecast potential risks, current capacity to cope with them is limited.

#### 6.2.3 Methods based on yield limiting factor

Yield prediction based on physiological and biochemical agronomic indicators, require frequent manual sampling, but achieving full spatial coverage is often challenging. In addition, many agronomic parameter-based approaches employ one or a few parameters for empirical modeling, which might lead to weaker generalizability.

Meteorological parameter-based methods predict potato yields under specific climatic conditions, without laborious field sampling. However, multi-year historical yield and climate data is difficult to obtain in reality. As with the agronomic parameter-based approach, which employs an empirical approach to modeling, this method also oversimplifies. Simulation results are not reliable with drastic changes in weather conditions. Providing farmers with suitable agricultural insurance may be a way to mitigate the adverse effects of unexpected weather conditions. With the growing scope of agricultural informatization, developing a precise and comprehensive agricultural information data platform containing a range of meteorological and yield data is crucial to achieve multi-year yield prediction.

Yield prediction methods based on input-output theory necessitate multiple input parameters, which are frequently derived from interviews with growers. Meanwhile, farmer surveys are also labor-intensive. Ensuring the accuracy of the data collected can be challenging (Fermont and Benson, 2011).

### 6.3 Uncertainties of yield prediction

Uncertainty is a range centered on the true value, and the larger the range the greater the uncertainty (Lu, 2004). Model uncertainty emerges due to necessary simplification of the real physical process. Differences between the theoretical and real values due to various factors such as assumptions made during model construction, boundary conditions, and the difficulty of reflecting them in calculations at the current state of technology are considered model uncertainty (Xing and Guo, 2006). Potato yield formation is a complex system determined by a combination of cultivation management practices, climatic conditions, soil conditions, and varieties. The yield simulation process could be influenced by any changes in the above conditions. At the same time, different parameter selections, model inputs, and model structures can lead to uncertainty when constructing potato yield prediction models. It is important to address sources of uncertainty and improve the adaptability of model prediction accuracy and prediction confidence for accurate and reliable potato yield prediction (Ma et al., 2021).

The causes of uncertainty include inherent limitations in predictability (e.g., future greenhouse gas emissions) and deficiencies in forecasting skills (e.g., flaws in model design)(Challinor et al., 2013). Model structure, inputs, and parameters are the three main sources of uncertainty (Wallach et al., 2016). Although the empirical statistical model is computationally simple, there is uncertainty in the functional form and coefficients of different modeling approaches (Wu et al., 2014). Any simple model-based or empirical regression-based inversion is unlikely to produce stable inversion results, and accounting for errors in ground truth and sensor data can improve the estimation accuracy of the parameters in the model (Fermont and Benson, 2011). The mechanistic model simplifies the real growth state of the crop due to assumptions made in the construction process, boundary conditions, and the difficulty of responding in the calculations at the current level of technology. Therefore, the uncertainty caused by the model structure needs to be considered in yield prediction.

Moreover, the accuracy and representativeness of the yield prediction model largely depends on the accuracy of the input data, including weather, soil, and management information. The uncertainty of these input data, especially in large-scale applications, can lead to significant errors in the prediction results. Therefore, it is necessary to further improve the accuracy of input data acquisition and data processing methods to improve the accuracy of yield prediction methods. In terms of RS, its integration with other methods, such as ground-based sensors and machine learning algorithms, can also improve the accuracy and practicality of yield prediction models. For example, RS data noise and environmental stress can increase the prediction uncertainty (Ma et al., 2021).

Uncertainty in model parameters, on the other hand, is a bias in simulation results due to deviations in the selection of parameters. For example, the impact of parameter value uncertainty on spring wheat phenology prediction uncertainty was quantified, and the relative contribution of model structure-driven and parameter value-driven uncertainty to overall prediction uncertainty were assessed (Alderman and Stanfill, 2017).

Few studies address all three sources of uncertainty simultaneously. For CGM, most studies have been devoted to the resolution of model input uncertainty. Sensitivity analysis is the most used uncertainty analysis method to determine which model inputs are more important for simulation results (Matott et al., 2009). In addition, other methods such as Monte Carlo analysis, Bayesian methods, and Generalized Likelihood Uncertainty Estimation (GLUE) can be used for model uncertainty analysis. Currently, most uncertainty assessments focus on the three staple crops wheat, maize, and rice, with less research on potato. Yield forecasting is the most concentrated area of uncertainty research because of its importance and because the results are influenced by many factors (Chapagain et al., 2022).

### 6.4 Fusing multi-source information

The yield estimation performance of models with a single source of estimation information are often inferior to estimation models that combine data from multiple sources. Previous studies have shown that combining multiple image feature parameters can improve yield prediction accuracy. Incorporating various parameters in RS yield estimation models can improve model performance. The crop model AquaCrop was combined with an economic model for optimizing irrigation management at the farm level (Garcia-Vila and Fereres, 2012). It is significant to use field trials, simulations, and deep learning models to study changing sowing dates to mitigate the effects of climate change (Dewedar et al., 2021). By combining deep learning algorithms with multi-source imagery including LiDAR and optical sensors, crop detection can be significantly improved (Prins and Van Niekerk, 2020). Coupling crop models with RS data is now a common method for yield estimation. RS images can be used to invert the LAI and applied to the crop model. However, the inconsistent relationship of LAI with most VIs and saturation problems lead to uncertainty in yield estimation. Other canopy variables, such as CC can be used in the AquaCrop model (Steduto et al., 2009) for yield prediction instead of LAI.

## 7 Conclusion

In this paper, methodologies for potato yield prediction and its evolution were comprehensively reviewed. The advantages and disadvantages of various strategies for potato yield prediction were compared. Moreover, the uncertainties of models and multi-source data fusion for yield prediction were discussed, providing a foundation for future studies.

Currently, potato yield prediction on large farmlands commonly employs RS and CGM. RS-based methods obtain farmland image information quickly and comprehensively by making full use of the unique advantages of RS platforms and sensors, however it is difficult to reflect the intrinsic mechanism of crop growth. CGM-based methods simulate the yield formation process of the crop, but their operation is challenging due to the lack of spatial expansion. In addition, methods based on agronomic parameters, meteorological parameters, and input-output theory are also widely used in the field of potato yield prediction.

With the progress in RS platforms, sensor technologies, and AI algorithms, UAVs and satellites equipped with advanced sensors have become mainstream tools for field monitoring and yield prediction at regional scales, which can be used for resource allocation and trade decisions. In addition, with the incorporation of modules, including water, nitrogen, meteorology, and economics, mechanisms of CGM have been more comprehensive. Combined with the comparison and improvement of multi-model ensembles, potato models are continuously improved in terms of precision management.

Multi-source and time-series data have great potential for future yield prediction, despite the current study using a limited number of varieties and sample sizes for potato yield prediction. In the future, it is necessary to pay attention to large time-series data studies with multiple varieties and large sample sizes.

## Author contributions

YL, JL and BL conducted the literature survey and drafted the article; CB, GL, SD, YY, SL, DL and LJ gave valuable comments to the manuscript and carried out critical revisions. JL and CB obtained the funding to support this study. All authors contributed to the article and approved the submitted version.
